# ROS-mediated waterlogging memory, induced by priming, mitigates photosynthesis inhibition in tomato under waterlogging stress

**DOI:** 10.3389/fpls.2023.1238108

**Published:** 2023-08-28

**Authors:** Lifei Niu, Fangling Jiang, Jian Yin, Yinlei Wang, Yankai Li, Xiaqing Yu, Xiaoming Song, Carl-Otto Ottosen, Eva Rosenqvist, Ron Mittler, Zhen Wu, Rong Zhou

**Affiliations:** ^1^ College of Horticulture, Nanjing Agricultural University, Nanjing, Jiangsu, China; ^2^ Vegetable Institute, Jiangsu Academy of Agriculture Science, Nanjing, Jiangsu, China; ^3^ College of Life Sciences, North China University of Science and Technology, Tangshan, Hebei, China; ^4^ Department of Food Science, Aarhus University, Aarhus, Denmark; ^5^ Department of Plant and Environmental Sciences, University of Copenhagen, Taastrup, Denmark; ^6^ Division of Plant Science and Technology, College of Agriculture, Food and Natural Resources, University of Missouri, Bond Life Sciences Center, Columbia, MO, United States

**Keywords:** tomato, repeated waterlogging, priming, stress memory, H2O2, Trichoderma harzianum

## Abstract

With global climate change, the frequency and intensity of waterlogging events are increasing due to frequent and heavy precipitation. Little is known however about the response of plants to repeated waterlogging stress events. The aim is to clarify physiological regulation mechanisms of tomato plants under repeated waterlogging stress, and whether *Trichoderma harzianum* can alleviate waterlogging injury. We identified two genotypes of tomato, ‘MIX-002’ and ‘LA4440’, as waterlogging tolerant and sensitive genotypes, respectively, based on plant biomass accumulation. The two tomato genotypes were subjected to a waterlogging priming treatment for 2 days (excess water for 1 cm above substrate surface) followed by a recovery stage for 2 days, and then a second waterlogging stress for 5 days (excess water for 1 cm above substrate surface) followed by a second recovery stage for 3 days. Leaf physiological, plant growth parameters, and the expression of five key genes were investigated. We found that the two genotypes responded differently to waterlogging priming and stress in terms of photosynthesis, reactive oxygen species (ROS), and osmotic regulatory mechanisms. Waterlogging stress significantly increased H_2_O_2_ content of ‘MIX-002’, while that of ‘LA4440’ had no significant change. Under waterlogging stress, photosynthesis of the two genotypes treated with waterlogging priming returned to the control level. However, *Trichoderma harzianum* treatment during the second recovery stage did not show positive mitigative effects. The plants of ‘LA4440’ with priming showed lower peroxidase (POD) activity and proline content but higher H_2_O_2_ content than that without priming under waterlogging stress. Under waterlogging stress with priming as compared to without priming, *SODCC2* was downregulated in two tomatoes, and *AGR2* and *X92888* were upregulated in ‘MIX-002’ but downregulated in ‘LA4440’. Overall, the two tomato genotypes exhibited distinct photosynthetic, ROS and osmotic regulatory mechanisms responding to the waterlogging stress. Waterlogging priming can induce stress memory by adjusting stomatal conductance, sustaining ROS homeostasis, regulating osmotic regulatory substances and key gene expressions mediated by H_2_O_2_, and thus alleviate the damage on tomato photosynthesis when waterlogging reoccurred.

## Introduction

Tomato (*Solanum lycopersicum* L.) is an annual vegetable of the tomato genus in the Solanaceae family, which is one of the highest value vegetables in the world. The world tomato harvested area was 5,009,027 and 5167388 ha and world average tomato yield was 368,906 and 366,015 hg/ha in the year of 2020 and 2021, respectively (http://faostat.fao.org/). From 1994 to 2021, Asia accounted for 54.9% tomato production with China as the biggest producer (http://faostat.fao.org/). With the continuous deterioration of the global environment, ecosystem disorder, and water resources distribution imbalance, waterlogging is becoming more and more common, which had devastating effects on the growth and development of plants (Pan et al., 2021; Zhou et al., 2022a; Zhou et al., 2022b). The main tomato production provinces in China, e.g., Henan, Shandong, Hainan, and Jiangsu, suffered from concentrated or extreme precipitation, leading to excess water and a severe threat to tomato plants grown in the field. Together with irrational irrigation in greenhouse, tomato plants can easily be subject to waterlogging stress.

Photosynthesis is one of the most important life activity processes in plants (Hall and Rao, 1999), which is sensitive to abiotic stress including waterlogging. Photosynthesis and aerobic respiration of pepper plants were inhibited during waterlogging stress (Ou et al., 2011), while anaerobic respiration was intensified, and CO_2_ together with toxic substances accumulated, thus accelerating leaf senescence (Zhou et al., 2020). In addition, waterlogging stress can induce excessive reactive oxygen species (ROS) accumulation such as H_2_O_2_ and O_2_^•─^, leading to cell death and plant senescence (Pan et al., 2021). Although most plant species are sensitive to waterlogging stress, the damage caused by waterlogging stress can be alleviated by stress priming, which has been reported for example in wheat (Li et al., 2011; Wang et al., 2016; Feng et al., 2022), soybean (Agualongo et al., 2022), and tomato (Zhou et al., 2022a).

Priming can induce stress memory in plants in both the present generation and in offspring, which is a promising strategy for coping with abiotic stresses (Wang et al., 2017; Feng et al., 2022; Lukić et al., 2023). A series of physiological and morphological changes happened when plants were subjected to mild stress, which induced stress memory and enabled plants to respond quickly and better when stress occurred again (Bruce et al., 2007; Walter et al., 2013). Previous studies provided evidence for waterlogging memory in plants (Li et al., 2011; Wang et al., 2016; Agualongo et al., 2022; Feng et al., 2022; Zhou et al., 2022a). For instance, wheat suffering waterlogging during the vegetative stage can efficiently improve the tolerance of wheat during the reproductive stage to waterlogging (Li et al., 2011). Waterlogging priming relieved the damage of waterlogging stress in wheat by increasing antioxidant capacity and proteins in the ethylene biosynthesis pathway (Wang et al., 2016). Seven days of waterlogging priming for alleviated oxidative stress as indicated by low H_2_O_2_ content, lipid peroxidation and antioxidant enzyme activities in roots and leaves of soybean when waterlogging reoccurred (Agualongo et al., 2022). Waterlogging priming in parental wheat could significantly enhance the waterlogging tolerance in offspring wheat with no effect on its growth and development under normal conditions (Feng et al., 2022). Our previous study found that waterlogging priming played a positive role in inducing stress memory by alleviating the waterlogging damage to photosynthesis of both wild and cultivated tomatoes (Zhou et al., 2022a), where the underlying mechanisms remained uninvestigated.

ROS are a class of ubiquitous molecules, such as O_2_^•─^, H_2_O_2_ and **·**OH (Hoidal, 2001). H_2_O_2_ is considered as the main ROS involved in cell signaling, since it is relatively stable (Mhamdi and Van Breusegem, 2018). In plants, H_2_O_2_ functions as a signaling molecule that participates in multiple signaling pathways, abiotic stress responses, and programmed cell death (Mittler et al., 2004; Sun et al., 2019). For instance, H_2_O_2_ is involved in plant responses to hypoxia stress as a second messenger (Baxter-Burrell et al., 2002) and to repeated heat stress as a signaling molecule (Sun et al., 2018). In addition, H_2_O_2_ can alter the expression of genes regulating antioxidant enzymes and relevant transcriptional factors to maintain the redox homeostasis in plant cells (Neill et al., 2002). However, whether and how H_2_O_2_ plays a key role in waterlogging memory in tomato plants remained unclear.

The investigation of antioxidant enzymes and their related gene expression provides insights into the regulatory mechanisms of plants responding to abiotic stress. Superoxide dismutase (SOD), catalase (CAT) and peroxidase (POD) are important enzymes for the scavenging of ROS and synergistically work with non-enzymatic systems to protect against ROS damage to plant cells (Mehla et al., 2017). SOD can catalyze O_2_^•─^ to form H_2_O_2_, being considered as a key component of biological defense against oxidative stress (Arbona et al., 2008; Hasanuzzaman et al., 2020). H_2_O_2_ can be converted into H_2_O and molecular oxygen being catalyzed by CAT and POD, thus reducing the stress damage (Arbona et al., 2008; Hasanuzzaman et al., 2020). The expression of *SODCC1* and *SODCC2* was upregulated in 10-day-old seedlings of Brazilian *indica* rice under salt stress (Menezes-Benavente et al., 2004). CAT and POD convert H_2_O_2_ into O_2_ and H_2_O, and act together with SOD to keep ROS homeostasis (Hasanuzzaman et al., 2020). A previous study found that salt treatment significantly elevated the transcription level of the *CAT2* gene in tomato leaves (Awaly et al., 2020). Cheng et al. (2016) found that 2-Cys peroxisome (*2-CP*) interacted with an ascorbate-dependent pathway and autophagy, which was involved in tomato’s response to high temperature via removing H_2_O_2_ and lipid peroxides. Moreover, Zhang et al. (2017) found that the *ARG2* gene encoding arginase 2 in tomato was significantly enriched under salt stress being involved in the metabolism of arginine and proline. Zeng et al. (2021) found that waterlogging could increase the expression of photosynthetic related genes and enhance the photosynthetic capacity of peanut leaves. Nevertheless, how antioxidant enzymes and their relevant genes coordinately regulate the response, especially photosynthetic capacity of tomato plants to repeated waterlogging stress needs investigation.

*Trichoderma harzianum* is a fungus that can effectively alleviate the damage of abiotic stress on plants, such as drought (Mastouri et al., 2012), salt (Zhang et al., 2019) and waterlogging (Elkelish et al., 2020). *Trichoderma harzianum* can enhance the antioxidant capacity and drought tolerance of tomato seedlings (Mastouri et al., 2012). It can improve salt tolerance of cucumber seedlings by regulating antioxidant enzymes to improve ROS scavenging ability and maintain osmotic and metabolic homeostasis (Zhang et al., 2019). *Trichoderma harzianum* improved the waterlogging tolerance of tomato seedlings through maintaining antioxidant status, glucose metabolism and critical gene expressions (Elkelish et al., 2020). However, the effect of *Trichoderma harzianum* on the recovery ability and response mechanism of tomato plants to repeated waterlogging remained unclear.

In this study, two tomato genotypes with different waterlogging susceptibilities were first selected and identified. The two genotypes were treated with waterlogging priming and recover, then treated with waterlogging and recover again, where *Trichoderma harzianum* was treated in the second recovery period. Plant morphological, physiological, biochemical, and molecular regulatory mechanisms were investigated. Our hypotheses were that (1) tomato genotypes with different waterlogging susceptibilities may exhibit different response mechanisms to repeated waterlogging stress, (2) H_2_O_2_, as the signal molecule, can be induced by the waterlogging priming and mediated waterlogging memory, which improved the tolerance of tomato plants when waterlogging reoccurred, and (3) *Trichoderma harzianum* may enhance the recovery ability of tomato plants after repeated waterlogging. This study shed light on the regulatory mechanisms of tomato plants to repeated waterlogging stress from the perspectives of morphology, physiology, biochemistry, and gene expressions, which laid a foundation for improving waterlogging tolerance of plants.

## Results

### Selection of waterlogging-tolerant and sensitive tomato genotypes based on biomass accumulation

#### First-round screening of 27 tomato genotypes

When waterlogging occurred, the plant height of genotypes No. 1, 20-23 and 25-27 were significantly higher, while that of genotypes No. 3, 5, 6, 9, 13, 18, 19 and 24 were significantly lower than the control (**Supplementary Table S1**; **Supplementary Figure S1A**). Waterlogging did not induce significant difference in stem diameter of the 27 genotypes as compared with the respective controls (**Supplementary Figure S1B**). Regarding fresh weight of shoot, only genotype No. 24 significantly increased (21.4%), while genotypes No. 3-7, 10-13, 16, 19 and 23 significantly decreased than the control (**Supplementary Figure S1C).** The shoot dry weight of genotypes No. 7 and 24 were significantly higher, but genotypes No. 3-6, 10, 11, 13, 15, 16 and 19 were significantly lower than the control (**Supplementary Figure S1D**). The malondialdehyde (MDA) content of 10 genotypes (No. 2, 3, 8, 12, 16, 18, 20, 24-26) significantly increased as compared with the respective controls (**Supplementary Figure S1E**).

Three genotypes No. 1, 7 and 9 were selected as waterlogging-tolerant genotype candidates. The reason is that the shoot dry and fresh weight and MDA content of the three genotypes under waterlogging were not significantly different from the control, except for the shoot fresh weight of genotype No.7 (**Supplementary Figure S1**). By comparison, genotypes No. 3, 10 and 16 were regarded as waterlogging-sensitive genotype candidates. The plant height, shoot dry and fresh weight of genotype No. 3 under waterlogging significantly decreased by 34.5%, 45.2% and 46.8%, respectively, while the MDA content of genotype No. 3 under waterlogging significantly increased by 21.8% (**Supplementary Figure S1**). The shoot dry and fresh weight of genotypes No. 10 and 16 under waterlogging was significantly lower than the respective controls (**Supplementary Figure S1**).

#### Second-round screening of six tomato genotypes

**Figure 1:**
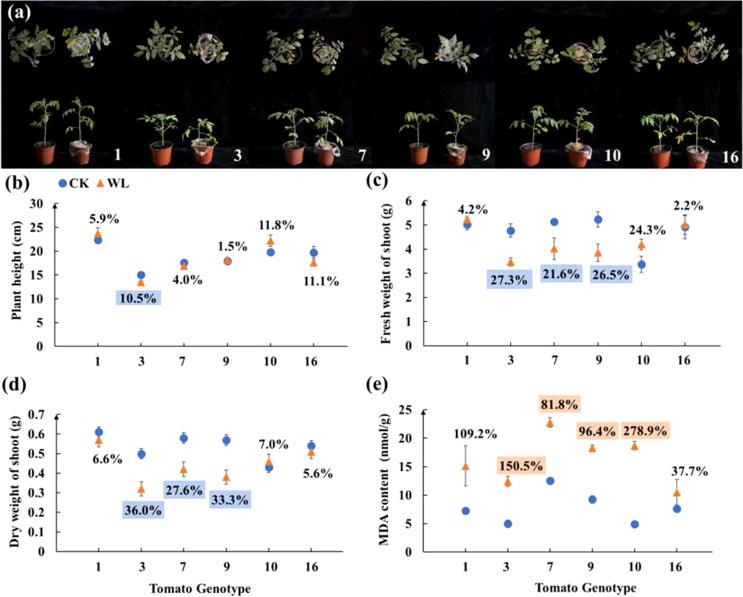
**(A)** Plant Phenotype, **(B)** plant height, **(C)** fresh weight of shoot, **(D)** dry weight of shoot, and **(E)** MDA content of six tomato candidates under CK and WL for 7 days The CK and WL indicated control and waterlogging treatment, respectively. The percentages referred to the increase/decreased percentages of the parameters in each genotype under waterlogging treatment as compared with the respective controls. The percentages above (orange square)/below (blue square) the marks indicated that the value of parameters significantly increased/decreased under waterlogging treatment as compared with the respective controls (*P* < 0.05). The percentages with no colors indicated no significant difference (*P* < 0.05).

The above six tomato candidates were further screened in the second-round screening. The genotype No. 1 showed no significant changes in terms of plant morphology, height, fresh and dry weight of shoot and MDA content (**Figure 1**). By contrast, the plants of genotype No. 3 showed significantly dwarf size under waterlogging as compared with the control (**Figure 1A**). After waterlogging, the plant height, fresh and dry weight of genotype No. 3 decreased by 10.5%, 21.6% and 27.6%, respectively, but the MDA content of genotype No. 3 increased by 150.5% (**Figures 1B–E**). Overall, genotypes No. 1 and No. 3 exhibited consistent waterlogging tolerance and sensitively, respectively, in both rounds of screening experiments.

### The responses of waterlogging-tolerant/sensitive tomato genotypes to repeated waterlogging in terms of leaf gas exchange, ROS homeostasis, key gene expressions, and biomass accumulation during WL (waterlogging) and R2 (the second recovery) stages

**Figure 2:**
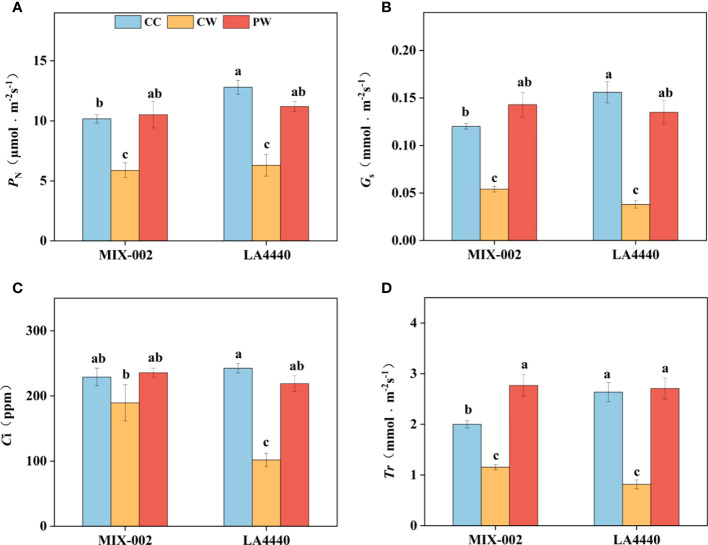
**(A)** Net photosynthetic rate (*P*_N_), **(B)** stomatal conductance (*G*s), **(C)** Intercellular CO_2_ concentration (*C*i), and **(D)** Transpiration rate (*T*r) of two tomato genotypes at the WL stage for 5 days The CC, CW and PW indicated Control + Control, Control + Waterlogging, and Priming + Waterlogging, respectively at the WL stage as shown in **Supplementary Figure S3**. Lowercase letters indicated ANOVA (analysis of variance) between different genotypes and different treatments (*P* < 0.05).

Waterlogging stress induced more decreases in photosynthetic parameters of ‘LA4440’ than that of ‘MIX-002’ (**Figure 2**). Net photosynthetic rate (*P*_N_), stomatal conductance (*G*s), intercellular CO_2_ concentration (*C*i), and transpiration rate (*T*r) of ‘MIX-002’ significantly decreased by 42.2%, 55.0%, 17.3% and 42.3%, respectively under CW as compared with that under CC (**Figure 2**). By comparison, the *P*_N_, *G*s, *C*i, and *T*r of ‘LA4440’ were significantly lower under CW than CC (50.8%, 75.6%, 58.0%, and 69.1%, respectively) (**Figure 2**). The *P*_N_, *G*s, *C*i, and *T*r of both genotypes significantly increased under PW than CW, except the *C*i of ‘MIX-002’ (**Figure 2**). Moreover, it is notable that the four photosynthetic parameters showed no significant difference in both genotypes between PW and CC, except the increased Tr of ‘MIX-002’ (**Figure 2**).

**Figure 3:**
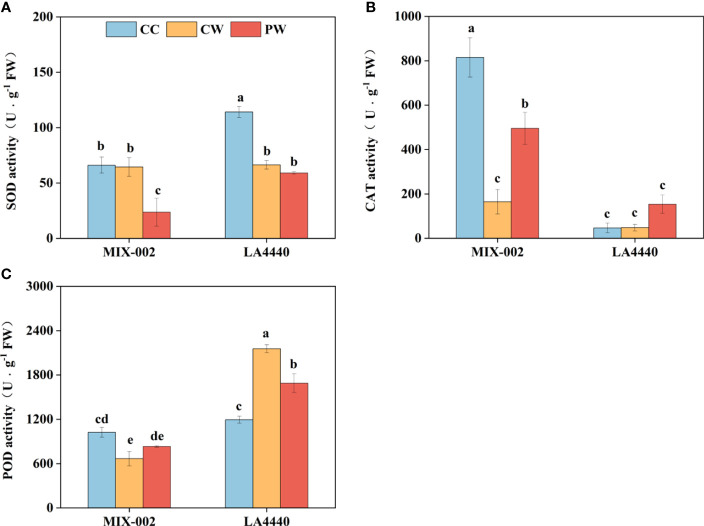
Activities of **(A)** superoxide dismutase (SOD), **(B)** catalase (CAT), and **(C)** peroxidase (POD) of two genotypes at the WL stage for 5 days The CC, CW and PW indicated Control + Control, Control + Waterlogging, and Priming + Waterlogging, respectively at the WL stage as shown in **Supplementary Figure S3**. Lowercase letters indicated ANOVA (analysis of variance) between different genotypes and different treatments (*P* < 0.05).

The SOD and POD activities of ‘LA4440’ were significantly lower and higher under CW than CC, respectively (**Figures 3A, C**). For ‘MIX-002’, the CAT and POD activities significantly decreased under CW than CC (**Figures 3B, C**). Priming induced lower SOD activity but higher CAT activity of ‘MIX-002’ as compared with CW (**Figures 3A, B**). The POD activity of ‘LA4440’ significantly dropped under PW as compared with CW (**Figure 3C**).

**Figure 4:**
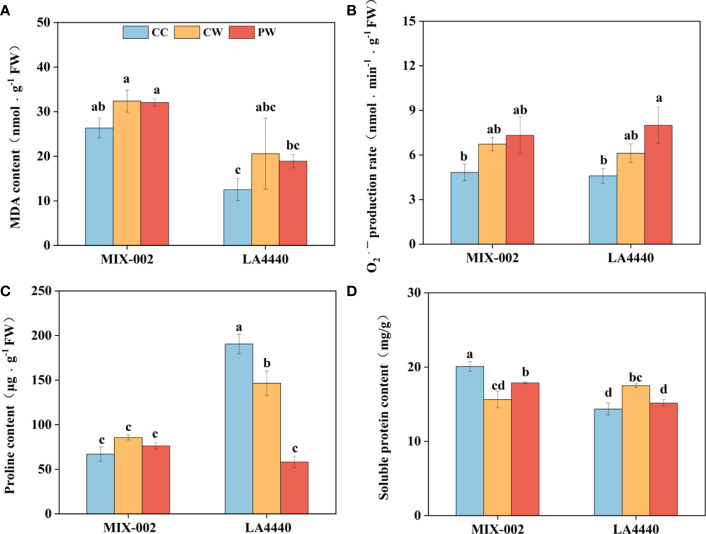
**(A)** Malondialdehyde (MDA) content, **(B)** O_2_^•─^ production rate, **(C)** proline content, **(D)** soluble protein content of two genotypes at the WL stage for 5 days The CC, CW and PW indicated Control + Control, Control + Waterlogging, and Priming + Waterlogging, respectively at the WL stage as shown in **Supplementary Figure S3**. Lowercase letters indicated ANOVA (analysis of variance) between different genotypes and different treatments (*P* < 0.05).

There was no significant difference between CW vs CC and PW vs CW in MDA content and O_2_^•─^ production rate of both genotypes (**Figures 4A, B**). However, the O_2_^•─^ production rate of ‘LA4440’ significantly increased by 74.5% under PW than CC (**Figure 4B**). Proline content of ‘LA4440’ significantly decreased by 23.0% under CW than CC, while that significantly decreased under PW as compared with both CC (69.4%) and CW (60.3%) (**Figure 4C**). Soluble protein content significantly decreased by 22.1% in ‘MIX-002’ but increased by 22.0% in ‘LA4440’ under CW as compare with the respective CC (**Figure 4D**). Priming induced a significant increase and decrease in soluble protein content of ‘MIX-002’ (14.3%) and ‘LA4440’ (13.3%), respectively, as compared with CW (**Figure 4D**).

**Figure 5:**
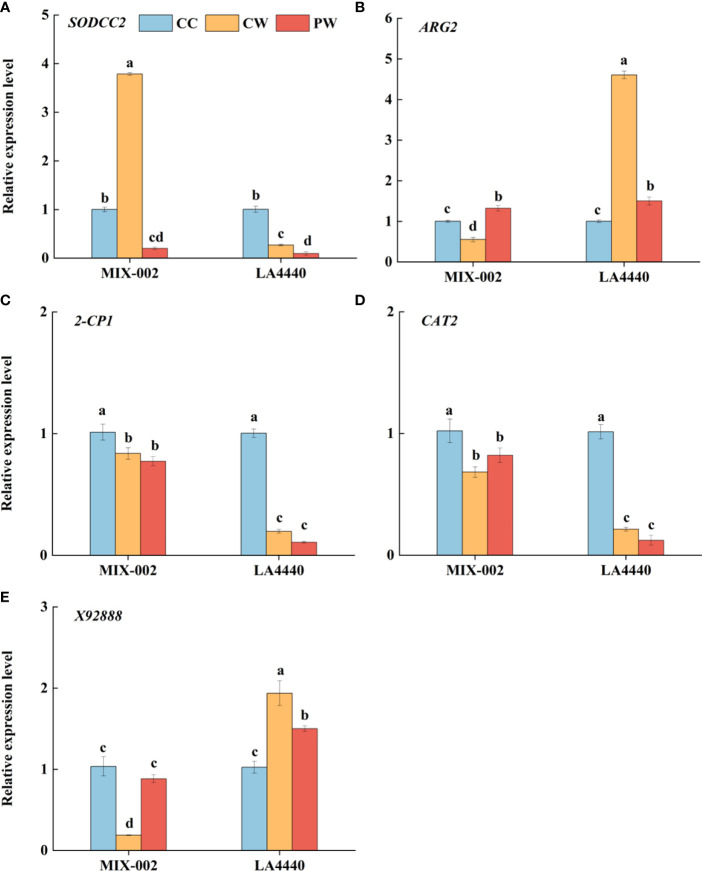
Expression level analysis of **(A)** *SODCC2*, **(B)** *ARG2*, **(C)** *2-CP1*, **(D)** *CAT2*, and **(E)** *X92888* of tomatoes at the WL stage for 5 days using qRT-PCR (quantitative real-time PCR) The CC, CW and PW indicated Control + Control, Control + Waterlogging, and Priming + Waterlogging, respectively at the WL stage as shown in **Supplementary Figure S3**. The *SODCC2*, *ARG2*, *2-CP1*, *CAT2*, and *X92888* indicated superoxide dismutase (SOD), reactive oxygen species (ROS), peroxidase (POD), catalase (CAT), and photosynthesis related genes. Lowercase letters indicated ANOVA (analysis of variance) between different genotypes and different treatments (*P* < 0.05).

The expression of *SODCC2* was significantly upregulated, but the expressions of *ARG2*, *2-CP1*, *CAT2* and *X92888* were significantly downregulated in ‘MIX-002’ under CW as compared with CC (**Figure 5**). Concerning CW vs CC in ‘LA4440’, the expressions of *ARG2* and *X92888* significantly increased but the expressions of the other three genes significantly decreased (**Figure 5**). The expression of *SODCC2* was significantly downregulated in both genotypes under PW as compared with CW (**Figure 5A**). By contrast, the expression of *ARG2* and *X92888* significantly increased in ‘MIX-002’, which significantly decreased in ‘LA4440’ under PW as compared with CW (**Figures 5B, E**).

**Figure 6:**
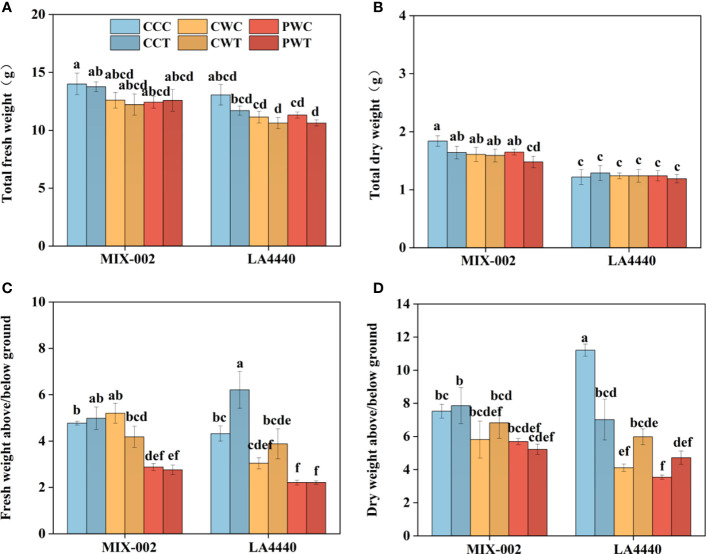
**(A)** Total fresh weigh, **(B)** total dry weight, **(C)** fresh weight above/below ground, **(D)** dry weight above/below ground of two tomato genotypes at the R2 stage for 3 days The CCC, CWC and PWC indicated Control + Control + Control, Control + Waterlogging + Control, and Priming + Waterlogging + Control, respectively at the R2 (the second recovery) stage as shown in **Supplementary Figure S3**. By comparison, CCT, CWT and PWT indicated the corresponding treatments with *Trichoderma harzianum*. Lowercase letters indicated ANOVA (analysis of variance) between different genotypes and different treatments (*P* < 0.05).

The size of plants under PW was smaller than CC and CW, demonstrating that *Trichoderma harzianum* did not show any alleviated effects on tomato phenotypes recovery (**Supplementary Figure S2A**). During the WL stage, the plant height of ‘LA4440’ under CW and PW showed no significant difference, which was significantly lower than CC (8.6% and 11.4%, respectively) (**Supplementary Figure S2B**). During the R2 stage, the plant height of ‘MIX-002’ significantly decreased under PWT as compared with CCC (**Supplementary Figure S2B**). In contrast, the plant height of ‘LA4440’ significantly decreased under CCT, CWT, PWC and PWT than CCC, where PWC and PWT showed the maximum deduction (**Supplementary Figure S2B**). There were no significant differences in stem diameter between all the treatments within each genotype in both WL and R2 stages (**Supplementary Figure S2C**). The shoot fresh weights of both genotypes were significantly lower under PWC and PWT than CCC (**Supplementary Figure S2D**). In contrast, the shoot fresh weights of both genotypes were significantly higher under PWC and PWT than CCC (**Supplementary Figure S2E**). By comparison, the shoot dry weight of ‘MIX-002’ was significantly lower under PWT than CCC, while the root dry weight of both genotypes showed no difference (**Supplementary Figure S2F, G**). The total fresh weight and dry weight of both genotypes showed no significant difference between all the treatments, except total dry weight of ‘MIX-002’ under PWT (**Figures 6A, B**). The decreased ratio of the shoot/root fresh weight under PWC and PWT than CCC was observed (**Figure 6C**). As compared with CCC, the ratio of the shoot/root dry weight significantly dropped under the other five treatments (**Figure 6D**).

### Trichoderma harzianum Leaf SPAD and H_2_O_2_ content in two tomato genotypes during the P, R1, WL and R2 stages

**Figure 7:**
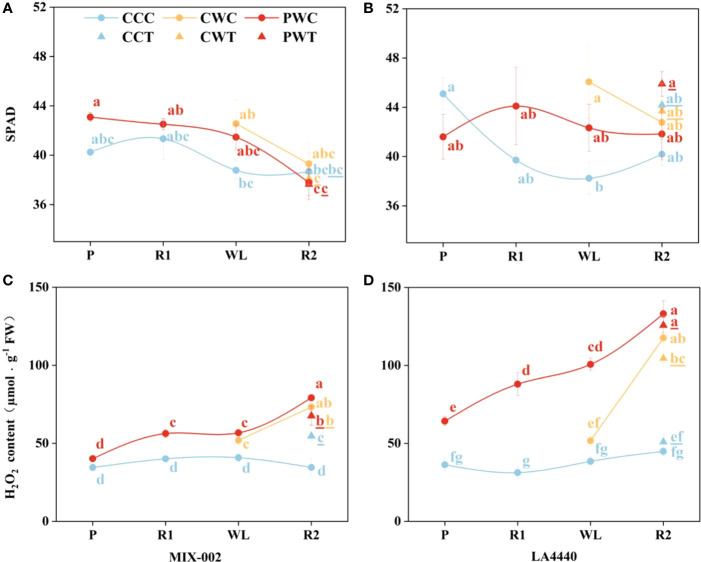
**(A, B)** SPAD, and **(C, D)** H_2_O_2_ content of ‘MIX-002’ and ‘LA4440’ of tomatoes at the stages of P for 2 days, R1 for 2 days, WL for 5 days and R2 for 3 days The stages of P, R1, WL and R2 showed the stages of waterlogging priming, the first recovery, waterlogging stress and the second recovery, respectively, corresponding to **Supplementary Figure S3**. The blue and red dots at the P and R1 stages indicated control and waterlogging priming, respectively. The blue, orange and red dots at the WL stage indicated CC (Control + Control), CW (Control + Waterlogging) and PW (Priming + Waterlogging), respectively. At the R2 stage, those treated with *Trichoderma harzianum* were marked as CCT, CWT and PWT, the lowercase letters of which was remarked with underlines; the CCC, CWC and PWC indicated those treated without *Trichoderma harzianum*. Lowercase letters indicated ANOVA (analysis of variance) between different genotypes and different treatments at all the four stages (*P* < 0.05).

The leaf SPAD (soil and plant analyzer development) is an important parameter to measure the relative content of chlorophyll in plants. The leaf SPAD of ‘MIX-002’ significantly decreased under PWC and PWT during the R2 stage as compared with PW during the P and R1 stages (**Figure 7A**). During the WL stage, the leaf SPAD of ‘LA4440’ was significantly higher under CW than CC (**Figure 7B**).

Generally, the H_2_O_2_ content of both genotypes under CCC kept constant (**Figures 7C, D**). Priming significantly increased the H_2_O_2_ content of ‘MIX-002’ during the R1 (40.4%), WL (30.0%) and R2 (129.1%) stages as compared the respective controls (**Figure 7C**). During the WL stage, the H_2_O_2_ content of ‘MIX-002’ significantly increased by 27.4% under CW as compared with CC (**Figure 7C**). During the R2 stage, the H_2_O_2_ content of ‘MIX-002’ significantly increased by 58.1%, 112.0%, 95.9%, 129.1%, and 95.6%, respectively, under CCT, CWC, CWT, PWC and PWT than CCC (**Figure 7C**). Moreover, priming significantly increased the H_2_O_2_ content of ‘LA4440’ during the P (77.1%), R1 (181.7%), WL (161.9%) and R2 (196.6%) stages as compared the respective controls (**Figure 7D**). During the R2 stage, the H_2_O_2_ content of ‘LA4440’ was significantly higher under CWC, CWT, PWC and PWT than CCC, with 162.2%, 132.9%, 196.6%, and 180.1% increased proportion, respectively (**Figure 7D**).

## Discussion

### Waterlogging tolerant/waterlogging sensitive tomato exhibited different physiological regulatory mechanisms in the presence of waterlogging

**Figure 8:**
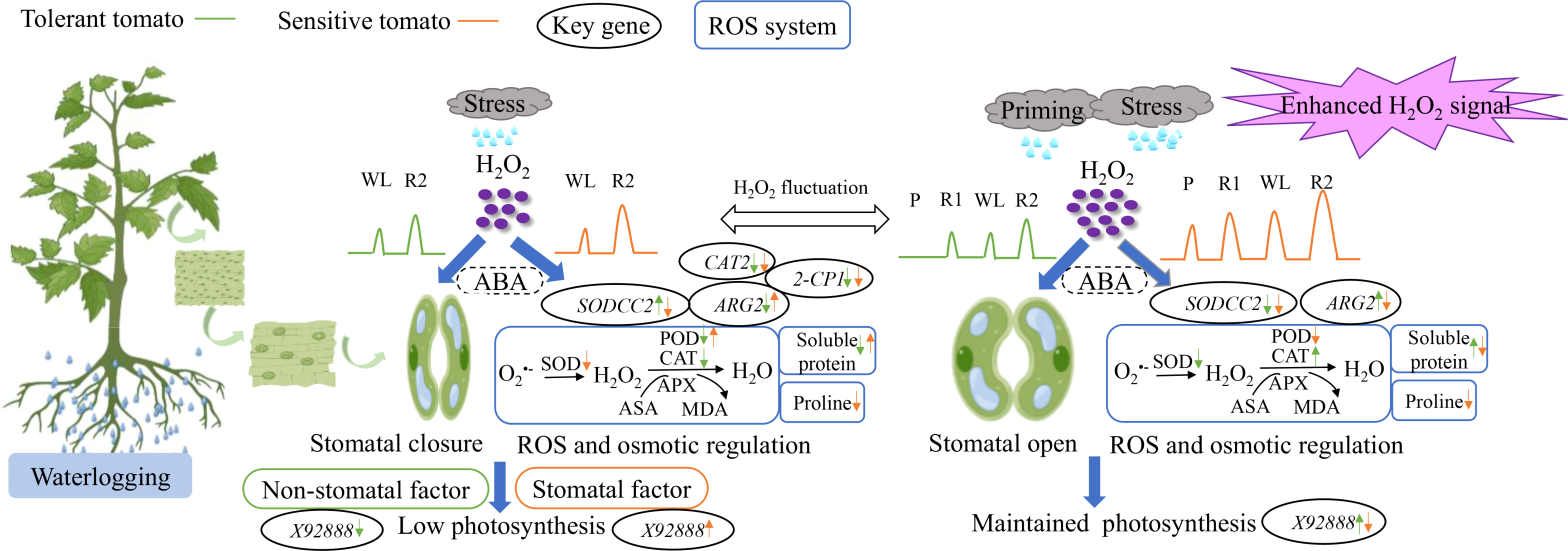
The effects of waterlogging without priming (left) and with priming (right) on tomato leaves Waterlogging stress without priming and with priming induced the distinct fluctuation of reactive oxygen species (mainly H_2_O_2_ here) in plant cells. Waterlogging stress without priming induced stomata closure as indicated by low *G*s and decreased photosynthetic capacity, while priming induced higher level of H_2_O_2_ as a signal to regulate stomata opening and maintain photosynthetic capacity. Left: tolerant and sensitive tomatoes under waterlogging stress exhibited different responses in terms of gas exchange, antioxidant system and osmotic regulatory mechanisms being associated with the alteration of the key gene expressions. Right: priming enhanced the regulation of stomatal conductance, antioxidant enzyme activities and osmotic regulatory substances mediated by H_2_O_2_. The coordinated function of ABA (abscisic acid) with H_2_O_2_ remained unexplored during waterlogging priming and stress in this study.

Waterlogging stress usually leads to chlorophyll degradation and decreased photosynthesis and ROS damage in plants (Pan et al., 2021). Waterlogging for three days significantly decreased the *P*_N_ and *G*s of pepper (Ou et al., 2011). In accordance, the *P*_N_ of waterlogging-tolerant and waterlogging-sensitive tomato significantly decreased by 42.2% and 50.8%, respectively under CW than CC (**Figure 2**). This indicated that the waterlogging stress inhibited photosynthesis of both genotypes being announced more in the sensitive genotype. Plant photosynthesis is often restrained due to stomatal and non-stomatal limitations (Flexas and Medrano, 2002). When *G*s and *C*i decrease simultaneously, the CO_2_ levels in plant cells cannot meet the needs of photosynthesis that retrained *P*_N_, being regarded as stomatal limitation (Farquhar and Sharkey, 1982; Zeng et al., 2021). By comparison, when *G*s drops but *C*i remains unchanged, it is the low activities of chloroplast and photosynthetic enzyme leading to the decreased *P*_N_, known as non-stomatal limitation (Farquhar and Sharkey, 1982; Zeng et al., 2021). Here, both genotypes showed a simultaneous decrease in *P*_N_ and *G*s, but only the waterlogging-sensitive tomato showed a significant decrease in *C*i under CW than CC (**Figure 2**). Therefore, the decreased photosynthesis of waterlogging-sensitive tomato under waterlogging stress was due to stomatal factors, while that of waterlogging-tolerant tomato was caused by non-stomatal factors (**Figure 8**). *X92888* (photosynthesis-related gene) was significantly downregulated in waterlogging-tolerant tomato but upregulated in waterlogging-sensitive tomato under CW as compared with CC (**Figure 5E**). Together, the two tomato genotypes exhibited different photosynthetic regulatory mechanisms in response to waterlogging stress, where the photosynthesis-related genes may play a critical role (**Figure 8**).

ROS is a normal product of plant cell metabolism, and insufficient O_2_ under waterlogging stress increases in intracellular ROS (Yan et al., 1996). MDA is the final decomposition product of membrane lipid peroxidation, caused by ROS with strong oxidative activities, which was applied to evaluate status of lipid peroxidation and reflect the degree of plant damage (Zhang F. et al., 2007). The MDA content and O_2_^•─^ production rate of both genotypes showed no significant difference between CC, CW and PW, except that the O_2_^•─^ production rate of the waterlogging-sensitive genotype was significantly higher under PW than CC (**Figures 4A, B**). This partially explained the sensitivity of ‘LA4440’ to waterlogging stress. Meanwhile, during the WL stage, waterlogging stress significantly increased the H_2_O_2_ content only in waterlogging-tolerant tomato (**Figures 7C, D**). The *ARG2* (ROS-related gene) was significantly downregulated in waterlogging-tolerant tomato but significantly upregulated in waterlogging-sensitive tomatoes under CW than CC (**Figure 5B**). Plants can rely on the antioxidant enzyme system to dynamically maintain the ROS balance and reduce the degree of oxidative damage under waterlogging stress (Zhang G. et al., 2007; Hasanuzzaman et al., 2020). We found that the SOD and CAT activity decreased only in sensitive and tolerant tomato, respectively, while the POD activity decreased and increased in tolerant and sensitive tomato, respectively, under CW than CC (**Figure 3**). The expression of *SODCC2* was significantly upregulated in tolerant tomato but downregulated in sensitive tomato under CW than CC (**Figure 5A**). The expression of *2-CP1* (POD-related gene) significantly decreased by 17.3% and 80.4%, and that of *CAT2* decreased by 33.1% and 78.9%, in tolerant and sensitive tomatoes under CW than CC (**Figures 5C, D**). Therefore, taking ROS and antioxidant enzymes changes into account, the ROS regulatory system of two tomato genotypes differentially responded to waterlogging stress (**Figure 8**).

In addition to antioxidant enzymes, plants can also alleviate stress damage by adjusting osmotic regulatory substances such as proline and soluble protein (Ru et al., 2022; Zulfiqar and Ashraf, 2022). We found that only the waterlogging-sensitive tomato showed lower proline content under CW than CC (**Figure 4C**). The waterlogging-tolerant tomato exhibited lower soluble protein content, but the waterlogging-sensitive had higher soluble protein content under CW than CC (**Figure 4D**). Therefore, waterlogging tolerant and sensitive tomatoes under excess water exhibited differences in gas exchange, antioxidant system and osmotic regulatory mechanisms, and these differences are related to changes in crucial gene expression levels (**Figure 8**).

### Waterlogging priming induced waterlogging memory associated with H_2_O_2_ in tomato when stress reoccurred

The root fresh weight was enhanced in primed plants than non-primed plants at the WL stage (**Supplementary Figure S2E**). The possible reason for this could be that more sugars were transported to roots, which provided a metabolic substrate for anaerobic respiration and thereby produced more ATP to maintain the root growth in primed plants than non-primed plants (Feng et al., 2022). Even though there were no positive effects of priming on shoot weight (**Supplementary Figures S2D, F**), it is worth noting that the *P*_N_, *G*s, *C*i and *T*r of both genotypes were significantly higher under PW than CW, except the *C*i of the tolerant genotype (**Figure 2**). The *P*_N_, *G*s, *C*i and *T*r of both genotypes did not show significant difference between PW and CC, except the *T*r of the tolerant genotype (**Figure 2**). These indicated that the priming effectively alleviated the stress damage on photosynthesis when waterlogging reoccurred, which was consistent with the previous findings on wheat (Wang et al., 2016) and tomato (Zhou et al., 2022a). Basal heat tolerance refers to the heat tolerance of plants without adaptation or pre-adaptation; pre-exposing plants to appropriate levels of heat stress enhances heat tolerance of plants, which is called acquired heat tolerance (Mittler et al., 2012; Stief et al., 2014). If the plants can memorize the high temperature and the acquired heat tolerance can be maintained for a long time, it is called maintenance acquired heat tolerance (Stief et al., 2014; Sun et al., 2018). Similarly, we found that both tomato genotypes showed maintenance acquired waterlogging tolerance by memorizing the stress during the process waterlogging priming.

In plants, ROS can induce acclimatization, defense, and memory of stress (Mittler et al., 2022). Waterlogging priming significantly induced the processes of stress defense and energy metabolism to increase the waterlogging tolerance of wheat (Wang et al., 2016). On one hand, as a non-radical form of ROS, H_2_O_2_ can cause oxidative stress by inactivating enzymes in plants (Gill and Tuteja, 2010). Agualongo et al. (2022) suggested that waterlogging priming decreased H_2_O_2_ content, lipid peroxidation, and activity of antioxidant enzymes and thereby alleviated oxidative stress when soybean suffered the waterlogging again. On the other hand, H_2_O_2_ is an important signaling molecule of different regulatory or enzymatic targets in plant cells (Mittler et al., 2004; Stone and Yang, 2006; Mhamdi and Van Breusegem, 2018). For waterlogging-tolerant tomato, primed plants showed no difference in H_2_O_2_ content during the P stage, but exhibited significantly higher H_2_O_2_ content than the respective controls during the R1, WL, and R2 stages (40%, 30%, and 129%, respectively) (**Figure 7C**). By comparison, priming significantly increased the H_2_O_2_ content in waterlogging-sensitive tomato as compared with the respective controls, with 77.1%, 181.7%, 161.9%, and 196.6% increased proportion during the P, R1, WL, and R2 stages, respectively (**Figure 7D**). Sun et al. (2018) concluded that during the recovery from heat stress, tomato seedlings upregulated H_2_O_2_ content and enhanced the expression of heat responsive genes to improve the maintenance of acquired heat tolerance. Together with our findings, waterlogging priming induced waterlogging memory by upregulating the H_2_O_2_ content as a stress signal for stress defense induction, and a high H_2_O_2_ level was maintained when waterlogging reoccurred, resulting in enhanced maintenance of acquired waterlogging tolerance especially in sensitive tomato (**Figure 8**).

We suggest that the high H_2_O_2_ level as a signal could enhance the regulation of stomatal conductance, antioxidant enzyme system and osmotic regulatory substances in tomato plants. Firstly, the *G*s of the two tomato genotypes with priming were significantly higher than unprimed plants (**Figure 2B**). An et al. (2008) found that H_2_O_2_ was an important signal of ABA-induced stomatal closure in *Vicia faba*, where exogenous ABA stimulated extracellular copper-containing diamine oxidases (CuAO) activity in guard cells, increased H_2_O_2_ production and [Ca^2+^] cell level and induced stomatal closure (An et al., 2008). However, the upregulation of H_2_O_2_ induced by waterlogging priming may act as a signal to regulate stomatal aperture through ABA in tomato plants. Secondly, waterlogging priming altered the activity of antioxidant enzymes mediated by H_2_O_2_ in two tomato genotypes. The SOD activity was downregulated, while CAT activity was upregulated, resulting in stable H_2_O_2_ level in tolerant tomato under PW than CW (**Figures 3**, **7**). By comparison, the POD activity of primed plants was significantly lower but the H_2_O_2_ level of primed plants was significantly higher than non-priming plants in sensitive tomato under waterlogging stress (**Figures 3**, **7**).

Thirdly, the contents of proline and soluble protein can reflect the osmotic regulation ability of plants under stress (Shahnaz et al., 2011; Kavi Kishor and Sreenivasulu, 2014). In this study, the proline content of waterlogging-sensitive tomato significantly decreased, while the soluble protein content of waterlogging-tolerant tomato significantly increased under PW than CW (**Figure 4**). In addition, ROS can trigger and modulate transcriptional regulation that enabled the plants to respond to stress and enhance plant resilience (Mittler et al., 2022). This explained why the sensitive tomato showed significantly decreased expression of *SODCC2*, *ARG2*, and *X92888* under PW than CW (**Figure 5**).

By regulating the distribution of metabolites, especially antioxidants, the redox states of plant cells can be balanced, thus achieving the regulation of metabolism and photosynthesis (Foyer and Shigeoka, 2011). We suggest that waterlogging priming enhanced the regulation of stomatal conductance, antioxidant enzyme activities and osmotic regulatory substances and key gene expressions mediated by H_2_O_2_. This contributed to keeping the ROS homeostasis, and promoting the maintenance of acquired waterlogging tolerance, thus alleviating the damage to tomato photosynthesis when waterlogging reoccurred (**Figure 8**). However, the application of *Trichoderma harzianum* did not show alleviated effects. The potential reasons could be that: 1) commercially available powder of *Trichoderma harzianum* applied might not work as well as the cultured strain; and 2) foliar spraying may not be a good approach to apply *Trichoderma harzianum* in waterlogging stress study. Thereby, suitable waterlogging priming could be beneficial to improve the waterlogging tolerance of tomato plants, while the application of *Trichoderma harzianum* needs further investigation to work.

## Materials and methods

### First-round screening of 27 tomato genotypes and second-round screening of six tomato genotypes

A total of 27 tomato genotypes were included in the first round of screening to evaluate their waterlogging susceptibilities. Among them, nine were provided by the Vegetable Physiological and Ecological Laboratory of Nanjing Agricultural University, and 18 were commercial varieties purchased from the market (**Supplementary Table S1**). Tomato seedlings were sown in 72-hole plates (54-cm length and 28-cm width) filled with a mixture of substrates (the volume ratio of turf, perlite, and vermiculite = 2:1:1). The seedlings were grown in a climate chamber (RGD-1000C, Ningbo, China) with a 14 h light time (8:00-22:00) and a 10 h nighttime (22:00-8:00). The light intensity was 30,000 lux and a daytime/night temperature was 25°C in the chamber. The 21-day-old seedlings with three fully expanded leaves were transplanted into pot (6.5 cm height and 6.5-cm diameter). The 28-day-old seedlings with four fully expanded leaves were treated under control (C) and waterlogging (WL). The WL treatment was conducted based on Zhou et al. (2022b), where the plants in the above small pots were put in big pots (10.8-cm height, 11.1-cm diameter) being filled with water for 1 cm above the surface of the substrate. There were five plants per treatment per genotype.

On the 7^th^ day of treatments, plant height, stem diameter, fresh and dry weight of shoots and leaf MDA content were measured with three biological replicates. The plant height was investigated by measuring the vertical distance from cotyledonary node to growing point using a ruler. The stem diameter (1 cm above the cotyledonary node) was measured using a vernier caliper. The plant was cut from the cotyledonary node and weighed to obtain the fresh weight of shoot. The shoot samples were put at 105 °C for 30 min followed by 80 °C for two days and weighed, which was the dry weight of shoot.

The third fully expanded leaf from top to bottom was selected to measure MDA content. The determination of MDA content was based on thiobarbituric acid (TBA) method (Kumar and Knowles, 1993) with minor modification. The 0.2 g fresh leaves were weighed, added to 5% trichloroacetic acid (TCA) solution, ground to homogenate, and then centrifuged. The supernatant was mixed with 67% TBA solution by oscillating. The samples were incubated in boiling water for 30 min. The 200 uL supernatant per sample was taken after quickly cooling and centrifuging. The samples were put on enzyme-labeled plate with three times of technical repetition. The absorbance of the solution under the wavelength of 450 nm, 532 nm and 600 nm were measured using Microporous plate detecting instrument (Cytation3, BioTek USA).

According to the results from the first-round screening, three waterlogging-tolerant tomato candidates (No. 1, 7 and 9) and three waterlogging-sensitive tomato candidates (3, 10 and 16) were selected for the second-round screening. The six tomato candidates were grown and treated in the same way as the first-round screening, where the same parameters were investigated in the same way as the first round of screening.

### The responses of waterlogging-tolerant and sensitive tomato plants to repeated waterlogging

#### Plant materials and experimental design

Based on the two rounds of screening, one waterlogging-tolerant genotype (No. 1, ‘MIX-002’) and one waterlogging-sensitive genotype (No. 3, ‘LA14440’) were selected. The aim was to clarify the response of tomato plants with distinct waterlogging susceptibilities to repeated waterlogging. Tomato seeds were sown, and the seedling were grown in climate chamber (RGD-1000C, Ningbo, China), with the same conditions as the above screenings. The 28-day-old seedings with four fully expanded leaves were treated under P, R1, WL and R2 stages (**Supplementary Figure S3**). The 2/3 plants were treated under control (C) and 1/3 plants were treated under waterlogging priming (P) during the P stage for two days. Then, all plants were treated under control during the R1 stage for two days of recovery. During the WL stage for five days, half plants from the previous 2/3 plants were still treated under control (CC, Control + Control), while the remaining half plants were treated under waterlogging stress (CW, Control + Waterlogging); the primed plants were treated with waterlogging stress (PW, Priming + Waterlogging). The plants under waterlogging priming and waterlogging stress were performed in the same way as the above screening experiment. Namely, the plants in the small pots were put in big pots being filled with water for 1 cm above the surface of the substrate. During the recovery stage, the big pots being filled with water were removed. During the R2 stage for three days, half of all the plants in each treatment (CC, CW and PW) were treated with *Trichoderma harzianum* (CCT, CWT, PWT), while the remaining half plants were under control (CCC, CWC, PWC) (**Figure 10**). The powder of *Trichoderma harzianum* (Beihai Qiangxing Biotechnology Co., LTD.) was made to the concentration of 8.33 mmol/L according to the instruction and then sprayed on the leaf surface every day. The spray lasted until the leaf surface was completely wet and the liquid started to drip downward. Equal amount of ddH_2_O was sprayed on the controlled plants. There were 24 plants per treatment per genotype. For all the measurements, there were three biological replicates. The third fully expanded leaves were used for the relevant measurements.

#### Measurements

Leaf photosynthetic parameters, antioxidant enzyme activities, MDA content, O_2_^•─^ production rate, proline, and soluble protein content were determined when the tomato plants were at the WL stage for five days.

The *P*_N_, *G*s, *C*i, and *T*r were measured using LI-COR Li-6400 portable photosynthesis system (LI-COR, Inc., Lincoln, NE, USA) at the WL stage for five days. The chamber conditions in LI-COR were set as follows: 25 ± 1 °C temperature, 300 μmol·m^-2^·s^-1^ light intensity, 500 μmol/s flow rate, 400 ± 10 μmol/L CO_2_ concentration.

The 0.2 g leaves (avoiding the main veins) were taken, washed and put in a pre-chilled mortar. Then, 0.05 mol/L pre-chilled phosphoric acid buffer (pH 7.8) was added, and the mixture was ground into a homogenate on an ice bath. The sample were centrifuged at 12000 rpm at 4 °C for 20 min, and the supernatant (extraction of the enzyme solution) was taken to determine the activities of SOD, POD, and CAT. The SOD activity was determined using nitrogen blue tetrazole (NBT) method (Zhou et al., 1997). The SOD activity unit was based on 50% inhibition of photochemical reduction of NBT as one enzymatic activity unit. The POD and CAT activity was determined using guaiacol method (Muñoz-muñoz et al., 2009) and spectrophotometry (Aebi, 1984), respectively. The POD and CAT activity units were calculated as one enzyme activity unit per min with 0.01 OD change. The activities of SOD, POD, and CAT were detected at 560, 470 and 240 nm, respectively, using UV-5500PC spectrophotometer (Metash, China).

The MDA content was measured in the same way as the section of screening. The O_2_^·─^ production rate was determined according to the method of Ke et al. (2007). The 0.05 M PBS (pH 7.8) and 10 mM hydroxylamine hydrochloride solution were added into the extraction of the enzyme solution obtained in the same way as the above. The extraction of the enzyme solution was replaced by PBS as control. The mixture was shaken well, kept at 25 °C for 1 h, then added with 17 mM p-aminobenzene sulfonic acid and 7 mM α-naphthylamine before vortex. Then, the samples were put at 25°C for 20 min, centrifuge at 3000 rpm for 3 min. The absorbance of the samples was measured at 530 nm using Microporous plate detecting instrument (Cytation3, BioTek, USA).

Proline content was determined using ninhydrin chromogenic method (Ábrahám et al., 2010). The 0.2 g leaf samples were weighed and mixed with 2 mL 3% sulfosalicylic acid dihydrate solution. The mixture was ground in mortar and transferred to a centrifuge tube, and then boiled for 10 min. The supernatant was taken after centrifuging the cooling samples and mixed with acetic acid and acidic ninhydrin. The mixture was boiled in a water bath for 30 min, and 2 mL toluene was added to the mixture after cooling. The upper layer of proline-toluene solution was gently pipetted into the colorimetric cup with a pipette, and toluene was used as control. The absorbance of the solution at 520 nm was measured using Microporous plate detecting instrument (Cytation3, BioTek, USA).

Soluble protein content was determined using coomassie brilliant blue G-250 staining method (Zhang et al., 2015). The enzyme solution was extracted in the same way as the extraction of the above enzyme solution for SOD. Then, 100 µL enzyme solution was added into 2.9 mL coomassie brilliant blue solution, and the absorbance was determined at 595 nm after reaction for 2 min using Microporous plate detecting instrument (Cytation3, BioTek, USA).

#### Gene expression pattern analysis

After extracting RNA from tomato leaves using TRIzol total RNA extraction reagent (Invitrogen, CA, USA), 1 µg qualified RNA samples were taken for first-strand cDNA synthesis. The reverse transcription PCR reaction was performed using reverse transcription kit (Thermo Fisher Scientific, Waltham, MA, USA). The reaction conditions were at 25°C for 10 min, 42°C for 15 min, and 85°C for 3 min. The expression patterns of SOD, ROS, POD, CAT, and photosynthesis related genes of plants at the WL stage for five days were analyzed using qRT-PCR (quantitative real-time PCR). The primers were designed using Primer 5, the information of which was shown in **Supplementary Table S2**. The amplification reactions were performed using LightCycler® 480 real-time PCR system by mixing the reagents as shown in **Supplementary Table S3**. The reaction procedure included pre-deformation at 95°C; for 60 s, denaturation at 95°C for 10 s, annealing at 60°C; for 30 s with 40 cycles.

#### Determination of leaf SPAD and H_2_O_2_ content

At the stages of P, R1, WL and R2, the leaf SPAD and H_2_O_2_ content under all the treatments were determined. The leaf SPAD data was acquired using SPAD-502 chlorophyll analyzer (Konica Minolta, Japan). Three points were randomly taken on each leaf escaping main vein, the average of which was calculated as one replicate of the SPAD value. The H_2_O_2_ content was determined based on potassium iodide spectrophotometry (Chakrabarty and Datta, 2008). The 0.2 g fresh leaf samples were ground in liquid nitrogen and centrifuged with 0.1% TCA at 3000 rpm for 20 min. The supernatant was mixed with 1 M KI solution and 100 mM potassium sulfate buffer solution. After the reaction in dark for 1 h, the absorbance at 390 nm was determined using Microporous plate detecting instrument (Cytation3, BioTek, USA) with 0.1% TCA as reference.

#### Plant harvest

At the WL stage for five days and the R2 stage for three days, plant height and stem diameter were measured in the same way as the screenings. At the R2 stage for three days, fresh and dry weight of shoot and root weight were determined. The shoot part of the plant was cut from the cotyledonary node and weighed to obtain the shoot fresh weight. The root was cleaned and weighed to obtain the root fresh weight. Afterwards, the samples were dried up at 105°C for 30 min followed by 80°C for two days and weighed, which was the dry weight.

### Statistical analysis

The t-test was applied to compare the results under control and waterlogging in the screening experiments (*P* < 0.05) using SPSS Statistics (version 25.0, IBM, USA). Analysis of Variance (ANOVA) were used to test the significant differences of physiological and metabolic data between the two genotypes under all the treatments (*P* < 0.05) using SPSS Statistics (version 25.0, IBM, USA). The relative expression of genes was calculated using the 2^-∆∆Ct^ method with *SLActin* as an internal reference. All the Figures were made using Microsoft Excel 2019 and Origin Pro 2022.

## Data availability statement

The original contributions presented in the study are included in the article/**Supplementary Material**. Further inquiries can be directed to the corresponding author.

## Author contributions

LN and RZ designed the experiment. LN and RZ analyzed the data and interpreted the results. LN, YL, and JY conducted the experiments. LN and RZ wrote the manuscript. The other authors contributed to the improvement of the manuscript. All authors contributed to the article and approved the submitted version.
