# Supplementary material for: ROS-mediated waterlogging memory, induced by priming, mitigates photosynthesis inhibition in tomato under waterlogging stress
Source: Front Plant Sci. 2023 Aug 28;14:1238108. doi: 10.3389/fpls.2023.1238108 (PMC10493394; doi:10.3389/fpls.2023.1238108)
Supplement: Supplementary file 1 [file DataSheet_1.docx]

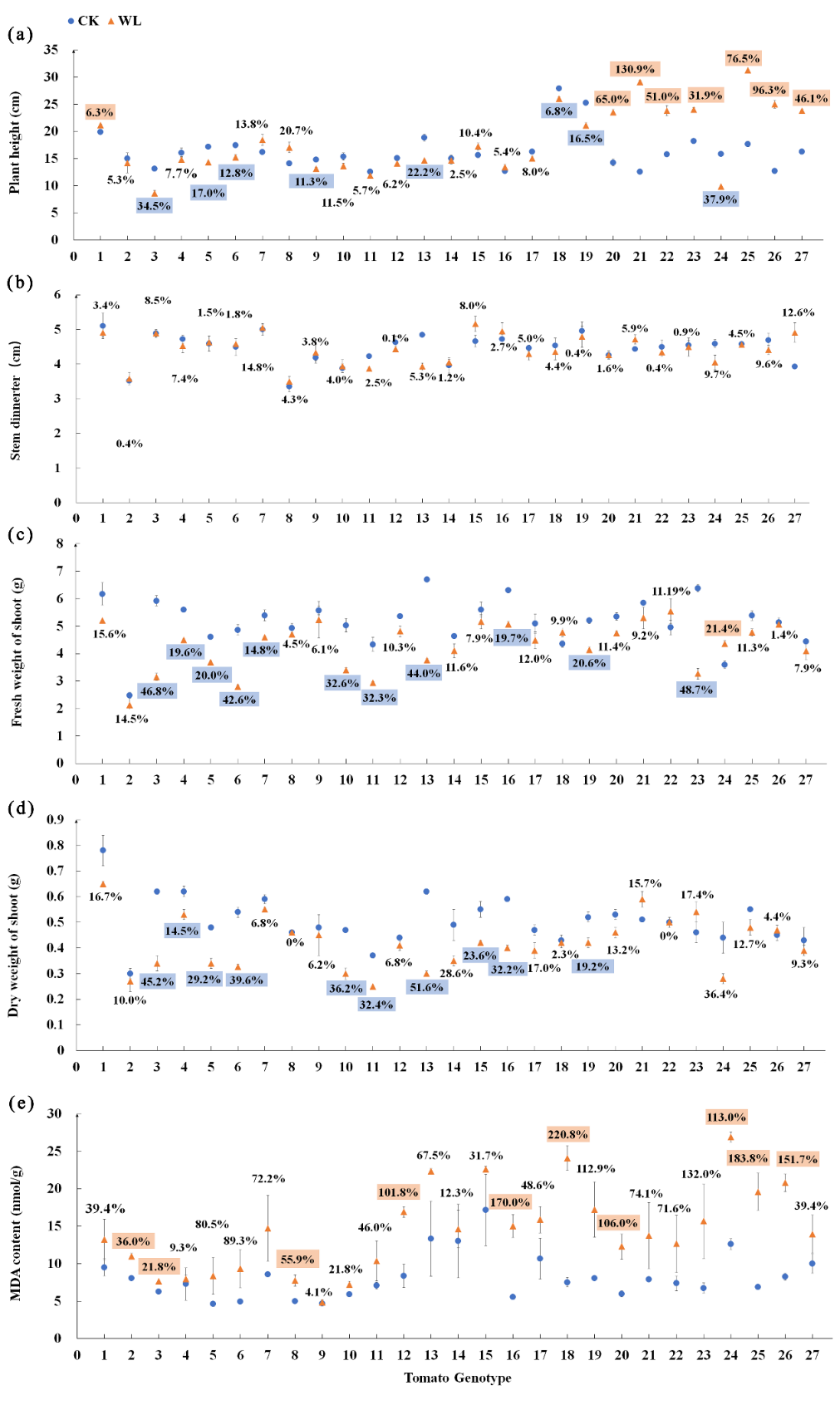
Figure S1. (a) Plant height, (b) stem diameter, (c) fresh weight of shoot, (d) dry weight of shoot, and (e) leaf MDA content of tomato genotypes under control and waterlogging stress for 7 days

The CK and WL indicated control and waterlogging treatment, respectively. The percentages referred to the increase/decreased percentages of the parameters in each genotype under waterlogging treatment as compared with the respective control. The percentages above (orange square)/below (blue square) the marks indicated that the value of parameters significantly increased/decreased under waterlogging treatment as compared with respective control (*P* < 0.05). The percentages with no colors indicated no significant difference (*P* < 0.05).


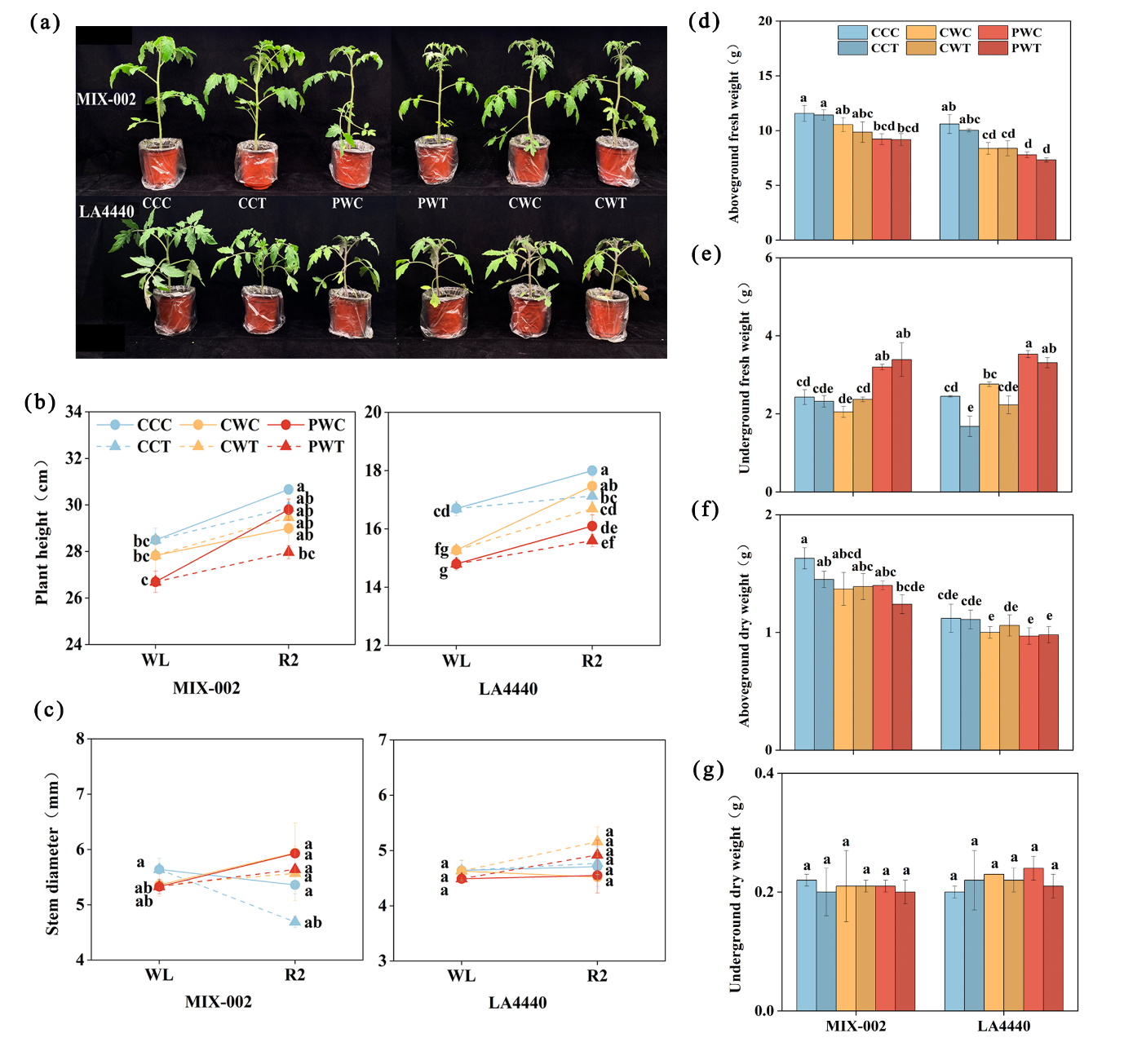
Figure S2. (a) Plant phenotype at the R2 stage for 3 days, (b) plant height and (c) stem diameter of two genotypes at the WL stage for 5 days and at the R2 stage for 3 days, (d) shoot (aboveground) fresh weight, (e) root (underground) fresh weight, and (f) shoot (aboveground) dry weight, and (g) root (underground) dry weight of two tomato genotypes at the R2 stage for 3 days.

The WL and R2 stages showed waterlogging stress and the second recovery stage, respectively, corresponding to Figure 9. The blue, orange and red dots in the WL stage indicated CC (Control + Control), CW (Control + Waterlogging) and PW (Priming + Waterlogging), respectively. At the R2 stage, those treated with *Trichoderma harzianum* were marked as CCT, CWT and PWT; the CCC, CWC and PWC indicated those treated without *Trichoderma harzianum*. Lowercase letters indicated ANOVA (analysis of variance) between different treatments within each genotype at the two stages (*P* < 0.05).


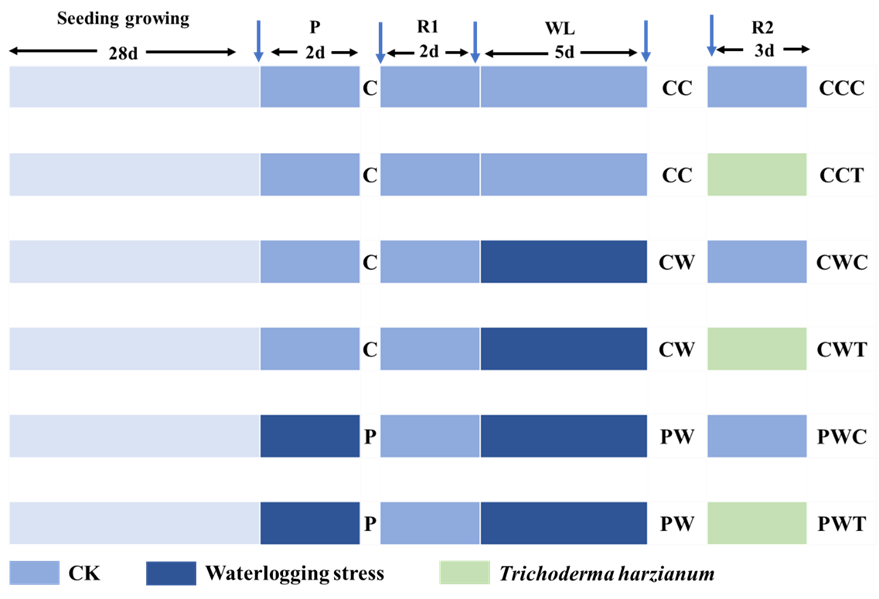
Figure S3. Experimental design to investigate the responses of two tomato genotypes to repeated waterlogging

The 28-day-old tomato seedlings were applied to the stages of P, R1, WL and R2, indicating the stages of waterlogging priming (2 days), the first recovery (2 days), waterlogging stress (5 days), and the second recovery (3 days), respectively. At the P and R1 stages, C and P indicated that the plants were under control and waterlogging priming, respectively. At the WL stage, CC, CW, and PW indicated Control + Control, Control + Waterlogging, and Priming + Waterlogging, respectively. At the R2 stage, those treated with *Trichoderma harzianum* were marked as CCT, CWT and PWT; the CCC, CWC and PWC indicated those treated without *Trichoderma harzianum*.

Table S1 Number, name, and source of the 27 tomato genotypes

| Number | Genotype name | Source |
| --- | --- | --- |
| 1 | MIX-002 | Vegetable Physiology and Ecology Laboratory, NAU |
| 2 | NT88 | Vegetable Physiology and Ecology Laboratory, NAU |
| 3 | LA4440 | Vegetable Physiology and Ecology Laboratory, NAU |
| 4 | Bulanni | Beijing Youfengna Agricultural Technology Co. |
| 5 | Chaoqun 968 | Beijing Youfengna Agricultural Technology Co. |
| 6 | Luode | Beijing Youfengna Agricultural Technology Co. |
| 7 | Fenbeibei F1 | Shouguang Xinxinran Gardening Co. |
| 8 | LA2093 | Vegetable Physiology and Ecology Laboratory, NAU |
| 9 | Hezuo 908 | Vegetable Physiology and Ecology Laboratory, NAU |
| 10 | Jinzhu | Taiwan Nongyou Seedling Co. |
| 11 | Alu 72-193 | Rexwan Agricultural Services Netherlands Ltd. |
| 12 | Jiaxiana | Rexwan Agricultural Services Netherlands Ltd. |
| 13 | Xiaoxia | Taiwan Nongyou Seedling Co. |
| 14 | Futesi | Rexwan Agricultural Services Netherlands Ltd. |
| 15 | Jinlingmeiyu | Jiangsu Jiang Vegetable Seedling Technology Co. |
| 16 | Ruifen 882 | Rexwan Agricultural Services Netherlands Ltd. |
| 17 | Motesi | Rexwan Agricultural Services Netherlands Ltd. |
| 18 | Fengshou | Rexwan Agricultural Services Netherlands Ltd. |
| 19 | Baili | Rexwan Agricultural Services Netherlands Ltd. |
| 20 | Zoufeiya | Rexwan Agricultural Services Netherlands Ltd. |
| 21 | Qianxi | Taiwan Nongyou Seedling Co. |
| 22 | Kaisa | Beijing Youfengna Agricultural Technology Co. |
| 23 | SVTG6210 | St. Nice Seeds (Beijing) Co. |
| 24 | Micro Tom | Vegetable Physiology and Ecology Laboratory, NAU |
| 25 | Jina | Vegetable Physiology and Ecology Laboratory, NAU |
| 26 | NC HS-1 | Vegetable Physiology and Ecology Laboratory, NAU |
| 27 | Tomato grape Jelly Bean Red | Vegetable Physiology and Ecology Laboratory, NAU |

The NAU indicated Nanjing Agricultural University, China.

Table S2 Sequence information of primers for qRT-PCR

| Gene | Forward primer (Sequence (5’-3’) | Reverse primer (Sequence (5’-3’) |
| --- | --- | --- |
| *SODCC2* | ACATACAAAAATGGTGAAGGCC | AGGATTGTAATGTGGTCCTGTT |
| *CAT2* | GCTGTCAAGTTTTACACCAGAG | GAACTTCATTCCATCACGGATG |
| *2-CP1* | ATTGGGTGTTTCTGTAGACAGT | AATTCAGATCACCTAGACCACC |
| *ARG2* | CCTCGCTTATGTAACCCTAAGT | AGAGTTAGAGAAAGAACGAGCC |
| *X92888* | TGAGTTTGAGCTAACTATGGCA | CTTCAAGTACATGCTCACAACC |
| *SLActin* | CTCTACATACTTGAGAGGTGCC | AGACGAGGAGAAAACATCACAA |

Table S3 qRT-PCR reaction system

| Reagent | Dosage /µL |
| --- | --- |
| Master Mix | 5 |
| cDNA | 1 |
| Forward primer | 0.4 |
| Reverse primer | 0.4 |
| ddH_2_O | 3.2 |
| Total Volume | 10 |
